# Two Measles Outbreaks After Importation — Utah, March–June 2011

**Published:** 2013-03-29

**Authors:** Mary Hill, Ilene Risk, Cindy Burnett, Wendy Garcia, Amy Carter, Lisa Guerra, Leona Goodsell, LaPriel Clark, Edward Redd, Shauna Nelson, Valoree Vernon, Karyn Leniek, Louise Saw, Jane F. Seward, Preeta K. Kutty, Greg Wallace, William J. Bellini, Paul A. Rota, Jennifer Rota, William A. Lanier

**Affiliations:** Salt Lake Valley Health Dept; Davis County Health Dept; Weber-Morgan Health Dept; Utah County Health Dept; Bear River Health Dept; Central Utah Public Health Dept; Utah Dept of Health; Div of Viral Diseases, National Center for Immunization and Respiratory Diseases; EIS Officer, CDC

Before licensure of a measles vaccine in 1963, more than 500,000 measles cases on average were reported in the United States each year during 1951–1962 (1). By 1993, through measles vaccination and control efforts, only 312 cases were reported nationwide (1). In 2000, the last year in which an outbreak had occurred in Utah, measles was declared “not endemic in the United States,” (2) but measles importations continue to occur, leading to outbreaks, especially among unvaccinated persons (3). Many U.S. health-care personnel have never seen a measles patient, which might hamper diagnosis and delay reporting. During March–June 2011, local health departments collaborated with the state health department in Utah to investigate two measles outbreaks comprising 13 confirmed cases. The first outbreak, with seven confirmed cases, was associated with an unvaccinated U.S. resident who traveled internationally; the second, with six confirmed cases, had an undetermined source. The genotype D4 sequences obtained from these two outbreaks differed by a single nucleotide, suggesting two separate importations. Health-care providers should remind their patients of the importance of being current with measles, mumps, and rubella (MMR) vaccination; this is especially important before international travel. Measles should be considered in the differential diagnosis of febrile rash illness, especially in unvaccinated persons with recent international travel. Reporting a confirmed or suspected case immediately to public health authorities is critical to limit the spread of measles.

## Outbreak 1

On April 5, 2011, a health-care provider notified the Salt Lake Valley Health Department (SLVHD) of an unvaccinated Salt Lake County resident aged 16 years with generalized rash (onset April 4) and a 3-day history of sore throat and fever (101.7°F [38.7°C]) (Table). When investigated by public health officials on April 6, the patient had a morbilliform rash, cough, coryza, conjunctivitis, and Koplik spots, and reported no recent travel or contact with ill persons. Serum collected on April 5 was positive for measles immunoglobulin M (IgM). On April 7, SLVHD announced that a measles case had been confirmed. On April 8, a health-care provider notified SLVHD that an unvaccinated Salt Lake County patient aged 15 years had sought care in late March with generalized rash (onset March 21), fever (103.7°F [39.8°C]), cough, coryza, and conjunctivitis. The patient had traveled in Europe during March 3–17. No measles testing was performed during the acute illness. Serum collected on April 8 was measles IgM-positive. This patient had attended a school class on March 21 with the patient reported previously. Five additional Salt Lake County residents were confirmed to have measles, with the last rash onset on April 17, 2011 (Figure).

## Outbreak 2

On May 24, 2011, a Cache County resident notified the Bear River Health Department that her unvaccinated child aged 7 years had signs and symptoms compatible with measles, including generalized rash (onset May 23) and fever (101.5°F [38.6°C]) (Table, Figure). She reported no recent travel outside Utah or contact with any person with a rash illness; the source was not identified. Serum collected on May 26 was measles IgM-positive. On May 31, the Bear River Health Department announced that a measles case had been identified. Two unvaccinated siblings of the patient, for whom the parents declined postexposure vaccination, were home-quarantined and developed measles, with rash onsets June 1 and 2, respectively. Additionally, two Cache County residents and one Millard County resident, all family members of the two siblings, were identified as having measles; the last reported rash onset was June 16, 2011.

During the two measles outbreaks, local health departments collaborated with the state health department in investigating and initiating active surveillance and outbreak response. Confirmed cases were defined using the 2010 Council of State and Territorial Epidemiologists measles case definition (4). Case-finding efforts included e-mail messages to health-care providers, press releases, syndromic surveillance for febrile rash illness, urgent-care facility admission data, and communications with hospital infection control practitioners. Laboratory methods included testing serum specimens for measles IgM and immunoglobulin G (IgG) at commercial and CDC laboratories, viral culture, polymerase chain reaction, genotyping, and testing for parvovirus B19 IgM. Public health investigators assessed patients and contacts for symptoms, vaccination history, and presumptive evidence of measles immunity.* Contacts without presumptive evidence of immunity were offered MMR vaccine or immunoglobulin, as appropriate, or placed in voluntary home quarantine.

In the two outbreaks, separated by 36 days, 13 persons were confirmed to have measles; nine (69%) were unvaccinated and had personal belief exemptions,† one had a documented history of 2 doses of measles antigen–containing vaccine, and three adult patients reported a history of vaccination, although vaccination records were not available (Table, Figure). Patients were aged 5–48 years. Measles genotype D4 was identified from the clinical samples obtained in both outbreaks (Table). Two unvaccinated patients were hospitalized; one for 3 days for respiratory complications and another overnight in an emergency department for observation. Among the 12 cases where the source of infection was known, measles infections were acquired during international travel (one case) and in households (eight) and schools (three). During the Salt Lake County outbreak, seven additional patients who initially tested positive or equivocal by commercial measles IgM testing were suspected of having measles but were not confirmed at CDC. Five of these had a history of vaccination, no direct epidemiologic link to the Salt Lake County cases, and were confirmed as parvovirus B19 cases by additional serologic testing. The sixth patient suspected of having measles was epidemiologically linked to the school and the seventh was a household contact of that patient; neither showed evidence of measles infection in samples tested by CDC.

For both outbreaks, approximately 13,000 contacts of patients were notified by visit, phone, letter, or e-mail. Health officials reviewed vaccination records of approximately 8,700 exposed persons, conducted 253 measles IgG antibody tests, and administered 484 MMR vaccine and 28 measles immunoglobulin doses as postexposure prophylaxis. Voluntary home quarantine of 192 exposed persons without presumptive evidence of immunity was requested.

### Editorial Note

Because measles remains endemic in many regions of the world, the United States continues to be at risk for measles importations and outbreaks. In 2011, a total of 220 measles cases were reported in the United States, the highest number of reported measles cases since 1996; 89% were associated with importations (2). The outbreaks in Utah and elsewhere during 2011 highlight the critical need for appropriate vaccination of U.S. residents, particularly those who travel internationally. The Salt Lake County outbreak began when an unvaccinated traveler from the United States developed measles on returning to the United States and infected four other unvaccinated persons.

The genotype D4 sequences obtained from the two Utah outbreaks differed by a single nucleotide. Each of the Utah sequences was identical to one of two predominant sequence variants of genotype D4 that were circulating in Europe during 2011 (5,6). This, together with the interval of 5 weeks without cases between the two outbreaks, suggests the second outbreak likely was the result of a separate importation from an unknown source, rather than a continuation of the first outbreak.

In the Salt Lake County outbreak, three of the patients were adolescents who acquired the disease in school. In 2010, an estimated 96.4% of children attending public school in Utah were vaccinated with 2 doses of MMR vaccine (7). The high level of vaccination coverage among schoolchildren likely helped contain this outbreak. None of the three patients infected by the index patient at school transmitted the disease to other students. Ensuring high vaccination rates among schoolchildren is important to limit measles transmission.

For patients with risk factors for measles (e.g., unvaccinated status, recent travel history, or known epidemiologic link to a confirmed measles case), health-care providers and public health officials should consider measles in the differential diagnosis of febrile rash illness and should consider other potential exposures, including parvovirus, when ordering laboratory tests. Because measles now occurs so rarely in the United States, interpretation of measles tests can be challenging, especially during outbreaks, and confirming and correctly classifying measles in vaccinated persons can be particularly difficult. False-positive measles IgM results might be obtained in response to infections caused by parvovirus (8,9) and other viruses, including enteroviruses, Epstein-Barr virus, and varicella zoster virus. The capture IgM assay methodology available at CDC’s Measles Virus Laboratory§ generally is less prone to nonspecific reactions; however, the low prevalence of measles in the United States results in a low positive predictive value regardless of the IgM assay used. Serum and respiratory specimens both should be collected from suspected patients at first contact, because serological testing coupled with molecular testing provides the best opportunity for laboratory confirmation (10).

What is already known on this topic?

Since introduction of the measles vaccine in 1963, the incidence of measles has declined significantly, such that measles is no longer endemic in the United States, and many U.S. health-care providers currently practicing have never seen a patient with measles. Measles remains common in parts of the world, however, and international travel–related outbreaks are becoming more common.

What is added by this report?

During March–June 2011, Utah investigated two measles outbreaks comprising 13 confirmed cases. One outbreak was associated with an unvaccinated U.S. resident who traveled internationally; the source was unknown for the second outbreak. Genotype D4 sequences obtained from the two outbreaks differed by a single nucleotide, suggesting separate importations; each of the sequences was identical to one of two predominant sequence variants of genotype D4 circulating in Europe during 2011.

What are the implications for public health practice?

Health-care providers should remind their patients of the importance of being current with measles, mumps, and rubella vaccination, especially before international travel. Recognition of suspected measles cases by health-care providers and immediate reporting to public health officials can help prevent illness and associated costs. False-positive measles serologic test results can be obtained in infections caused by parvovirus and other viruses, including enteroviruses, Epstein-Barr virus, and varicella zoster virus.

Measles cases and outbreaks can have considerable impact on communities in the United States and often require substantial resources for public health response. Recognition of suspected measles cases by health-care providers and immediate reporting to public health officials can help limit illness and associated costs. For the two Utah outbreaks combined, those costs were estimated from multiple sources to exceed $330,000 for public health personnel time at state and local levels, vaccine administration, laboratory testing, and outbreak control efforts. Unvaccinated persons put themselves and their communities at risk for measles. Maintaining high vaccination coverage and rapid public health response is critical to ensuring continued measles elimination in the United States.

## Figures and Tables

**FIGURE:**
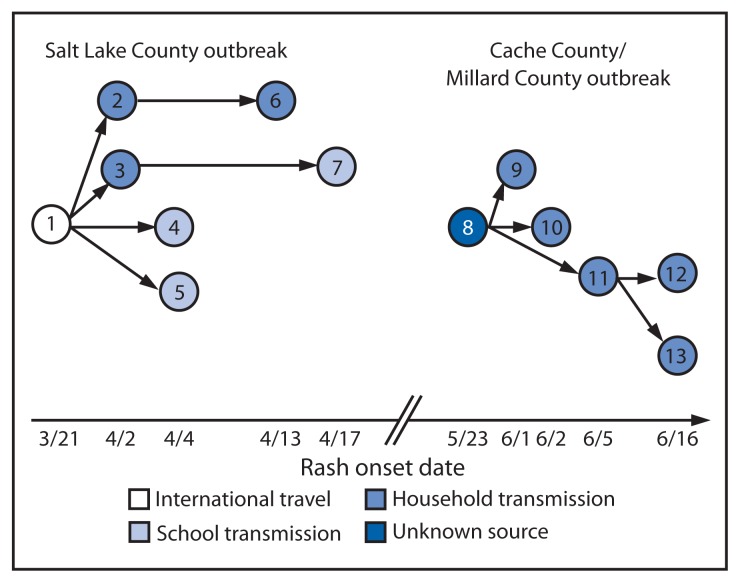
Timeline demonstrating chain of measles transmission in 13 cases, by date of rash onset and transmission setting — Utah, March–June 2011

**TABLE:** Characteristics of confirmed measles cases, by date of rash onset — Utah, March–June 2011

Location	Case no.	Date of rash onset	Age at onset (yrs)	Previous MMR vaccine doses	Symptoms				Transmission setting	Genotype	Total contacts*

					Fever	Cough	Coryza	Conjunctivitis			
**Salt Lake County**											
	1	3/21/2011	15	0	+	+	+	+	International travel	NA	**1,418**
	2	4/2/2011	18	0	+	+	+	+	Household	NA	**3,702**
	3	4/2/2011	12	0	+	+	+	+	Household	NA	**1,022**
	4	4/4/2011	18	0	+	+	+	+	School	NA	**98**
	5†	4/4/2011	16	0	+	+	+	+	School	D4	**2,100**
	6	4/13/2011	22	0	+	+	+	+	Household	NA	**1,409**
	7	4/17/2011	13	2	+	+	+	+	School	NA	**985**
**Cache County/Millard County**											
	8†	5/23/2011	7	0	+	+	+	+	Unknown	D4	**5**
	9	6/1/2011	11	0	+	+	+	+	Household	D4	**105**
	10	6/2/2011	5	0	+	+	+	+	Household	D4	**105**
	11	6/5/2011	44	1§	+	+	+	+	Household	NA	**405**
	12	6/16/2011	44	2§	−	−	+	+	Household	NA	**5**
	13	6/16/2011	48	1§	+	+	+	−	Household	D4	**905**

**Abbreviations:** MMR = measles, mumps, and rubella; NA = not available.

* Contacts are not mutually exclusive.

† Cases 5 and 8 were the first cases in the respective outbreaks to be reported to the local public health departments.

§ Vaccination history based on verbal reporting only.

## Footnotes

* Presumptive evidence of measles immunity is defined as documented receipt of 2 doses of live measles virus–containing vaccine, laboratory evidence of measles immunity, documentation of physician-diagnosed measles, or birth before 1957.

† Immunizations required – Exceptions – Grounds for exemption from required immunizations. Utah Code 53A-11-302 (2010). Available at http://le.utah.gov/~code/title53a/htm/53a11_030200.htm.

§ Additional information available at http://www.cdc.gov/measles/lab-tools/measles-virus-lab.html.
